# Dry and liquid formulations of IBT-V02, a novel multi-component toxoid vaccine, are effective against *Staphylococcus aureus* isolates from low-to-middle income countries

**DOI:** 10.3389/fimmu.2024.1373367

**Published:** 2024-04-03

**Authors:** Yu Wang, Ipsita Mukherjee, Arundhathi Venkatasubramaniam, Dustin Dikeman, Nicholas Orlando, Jing Zhang, Roger Ortines, Mark Mednikov, Shardulendra P. Sherchand, Tulasikumari Kanipakala, Thao Le, Sanjay Shukla, Mark Ketner, Rajan P. Adhikari, Hatice Karauzum, M. Javad Aman, Nathan K. Archer

**Affiliations:** ^1^ Department of Dermatology, Johns Hopkins University, Baltimore, MD, United States; ^2^ Integrated Biotherapeutics Inc., Rockville, MD, United States; ^3^ Center for Precision Medicine Research, Marshfield Clinic Research Institute, Marshfield, WI, United States; ^4^ Engineered Biopharmaceuticals, Danville, VA, United States

**Keywords:** vaccine development, Staphylococcus aureus, preclinical infection models, toxins, neutralizing antibodies, LMIC, IBT-V02

## Abstract

*Staphylococcus aureus* is the leading cause of skin and soft tissue infections (SSTIs) in the U.S. as well as more serious invasive diseases, including bacteremia, sepsis, endocarditis, surgical site infections, osteomyelitis, and pneumonia. These infections are exacerbated by the emergence of antibiotic-resistant clinical isolates such as methicillin-resistant *S. aureus* (MRSA), highlighting the need for alternatives to antibiotics to treat bacterial infections. We have previously developed a multi-component toxoid vaccine (IBT-V02) in a liquid formulation with efficacy against multiple strains of *Staphylococcus aureus* prevalent in the industrialized world. However, liquid vaccine formulations are not compatible with the paucity of cold chain storage infrastructure in many low-to-middle income countries (LMICs). Furthermore, whether our IBT-V02 vaccine formulations are protective against *S. aureus* isolates from LMICs is unknown. To overcome these limitations, we developed lyophilized and spray freeze-dried formulations of IBT-V02 vaccine and demonstrated that both formulations had comparable biophysical attributes as the liquid formulation, including similar levels of toxin neutralizing antibodies and protective efficacy against MRSA infections in murine and rabbit models. To enhance the relevancy of our findings, we then performed a multi-dimensional screen of 83 *S. aureus* clinical isolates from LMICs (e.g., Democratic Republic of Congo, Palestine, and Cambodia) to rationally down-select strains to test in our *in vivo* models based on broad expression of IBT-V02 targets (i.e., pore-forming toxins and superantigens). IBT-V02 polyclonal antisera effectively neutralized toxins produced by the *S. aureus* clinical isolates from LMICs. Notably, the lyophilized IBT-V02 formulation exhibited significant *in vivo* efficacy in various preclinical infection models against the *S. aureus* clinical isolates from LMICs, which was comparable to our liquid formulation. Collectively, our findings suggested that lyophilization is an effective alternative to liquid vaccine formulations of our IBT-V02 vaccine against *S. aureus* infections, which has important implications for protection from *S. aureus* isolates from LMICs.

## 1 Introduction

*Staphylococcus aureus* is a major health burden worldwide, being the predominant cause of skin and soft tissue infections (SSTIs), bacteremia, and pyomyositis (1–3). Furthermore, a global antibiotic resistance study found that methicillin-resistant *S. aureus* (MRSA) is now the major clinical isolate recovered in infected patients from both industrialized and low-to-middle income countries (LMICs) (4). This is particularly concerning in LMICs, which are disproportionately affected by antibiotic-resistant bacterial infections due to limited access to healthcare resources, decreased surveillance of antibiotic-resistance, and challenges to widespread vaccination distribution (5–9). For instance, in-hospital mortality due to intensive care unit-acquired infections and *S. aureus* bacteremia is significantly higher in LMICs than industrialized countries (10, 11). Thus, there is an unmet need for alternative therapeutic strategies to circumvent the emergence of antibiotic-resistant *S. aureus* infections, especially in LMICs.

We have developed IBT-V02 (12), a novel multi-component toxoid vaccine against *S. aureus* infections that neutralizes extracellular pore-forming toxins α-hemolysin (Hla), Panton-Valentine leukocidin (PVL), leukocidin AB (LukAB), and the superantigens toxic shock syndrome toxin-1 (TSST-1) and staphylococcal enterotoxins A and B (SEA and SEB). These toxins are important in *S. aureus* pathogenesis as they selectively kill or manipulate immune cells to evade host immunity (13, 14). This is especially pertinent as *S. aureus* cell-associated antigens have failed as vaccine targets in clinical trials (15–17). A liquid formulation of IBT-V02 was previously shown to have marked efficacy against *S. aureus* clinical isolates from the industrialized world (e.g., USA100, USA300, and USA1000) in a murine intradermal infection model (12). However, it is unclear whether dry formulations of IBT-V02 (e.g., lyophilized and spray freeze-dried), which may overcome the limitations of maintaining the cold chain and storage in LMICs (18), can recapitulate efficacy of the liquid IBT-V02 formulation. Furthermore, whether dry and liquid formulations have comparable efficacy against *S. aureus* clinical isolates from LMICs is unknown.

To address these gaps in knowledge, we first developed and biochemically tested the protein integrity, long-term stability, and immunogenicity of dry formulations of IBT-V02 in comparison to the liquid formulation. Moreover, we performed comparative efficacy studies of the dry and liquid formulations using a murine intradermal infection model and a novel rabbit pyomyositis infection model. Furthermore, we collected *S. aureus* clinical isolates from LMICs and used a rational down-selection strategy to identify isolates with broad toxin and superantigen production. Finally, we used the selected LMIC isolates to test the comparative efficacy of dry and liquid formulations of IBT-V02 in bacteremia and intradermal infection models in mice.

## 2 Materials and methods

### 2.1 Bacterial strains and cell lines

Bioluminescent *S. aureus* strain USA300 (SF8300) was kindly provided by Dr. Binh Diep at UCSF (19). *S. aureus* strains referred to as ‘Cambodia isolates’ were kindly provided by Dr. Catherine Moore at University of Oxford, UK. *S. aureus* strains referred to as ‘Kinshasa isolates’ were kindly provided by Dr. Frieder Schaumburg at University Hospital of Munster, Germany. *S. aureus* strains referred to as ‘Gaza isolates’ were kindly provided by Dr. Barry Kreiswirth at Center for Discovery & Innovation, HMH, NJ, USA.

The HL-60 cells, from ATCC (atcc.org), Manassas, VA were cultured in RPMI media containing 82 U/ml of penicillin and streptomycin with 16% FBS. Cells were differentiated in media with 1.5% dimethyl sulfoxide (DMSO), as previously described (20).

### 2.2 Preparation of inoculum for infection

For mouse infections with USA300 (SF8300) and LMIC isolates (KCO075, Pyo603, or Gaza209) and for rabbit infections with USA300 (SF8300), *S. aureus* strains were cultured in tryptic soy broth (TSB) as previously described (21, 22). Briefly, *S. aureus* was streaked onto a tryptic soy agar (TSA) plate (TSB plus 1.5% bacto agar (BD Biosciences)) and grown overnight at 37°C in a bacterial incubator. Two-to-three colonies were picked and cultured in TSB at 37°C in a shaking incubator (MaxQ HP 420, ThermoFisher) (240 rpm) overnight (16 h), followed by a 1:50 subculture at 37°C for 2 h to obtain mid-logarithmic phase bacteria. The bacteria were pelleted and washed in sterile phosphate-buffered saline (PBS). The absorbance at 600 nm (A600) was measured to estimate the number of colony-forming units (CFU) for inoculation, which was verified in each experiment after overnight culture on TSA plates.

### 2.3 Animals

For mouse experiments, 6-week-old female BALB/c mice obtained from Charles River were used. For rabbit experiments, 16-week-old female Dutch Belted Rabbits obtained from Robinson Services were used. All animals were maintained under the same specific pathogen-free conditions, with air-isolated cages at an American Association for the Accreditation of Laboratory Animal Care (AAALAC)-accredited animal facility at Johns Hopkins University and Integrated BioTherapeutics (IBT). They were handled according to procedures described in the Guide for the Care and Use of Laboratory Animals as well as Johns Hopkins University’s policies and procedures as outlined in the Johns Hopkins University Animal Care and Use Training Manual. Studies at JHU were approved by the Johns Hopkins Animal Care and Use Committee (Protocol #: MO21M378 for mice, Protocol#: RB22M400 for rabbit). Mouse studies at IBT were approved by institutional animal care and use committees (IACUC) (Immunogenicity Protocol# AP160805 and Efficacy against bacterial challenge Protocol#: AP-161007).

### 2.4 Vaccination protocol

Mice and rabbits were immunized intramuscularly (IM) or subcutaneously (SC) on each side of the tail base or thigh muscle (50 µl each side) three times, two weeks apart, with a total of 50 µg of IBT-V02 (10 µg each antigen) or 50 µg of BSA in 200 µg Alhydrogel in 100 µl histidine-borate buffer (buffer reconstituted in sterile water in the case of lyophilized vaccine) for each immunization. For mouse serological analyses, mice were bled via the retro-orbital (RO) route prior to and 10 days after the final immunization. For rabbit serological analyses, rabbits were bled via the central ear artery prior to, 10 days after the final immunization, and 7 days after infection.

### 2.5 Intradermal infection model in mice

Two weeks after the last immunization, the ID infection was performed as previously described (12, 23, 24). Briefly, the dorsal backs of anesthetized mice (2% isoflurane) were shaved and intradermally injected with *S. aureus* (8×10^6^ CFU of SF8300, 1×10^9^ CFU of KCO075, 3×10^8^ CFU of Pyo603, 2×10^8^ CFU of Gaza209) in 100 µl of PBS using a 29-gauge insulin syringe. Inocula were chosen to achieve an ~2 cm^2^ lesion size for each *S. aureus* isolate. Total lesion size (cm^2^) was measured by analyzing digital photographs using Image J ((https://imagej.nih.gov/ij/) and a millimeter ruler as a reference. For the mice infected with bioluminescent SF8300, *in vivo* bioluminescence (BLI) was performed using a Lumina III IVIS (PerkinElmer) and total flux (photons/s) was measured within a 1.78×1.78 cm circular region of interest using Living Image software (PerkinElmer). Ex vivo CFU were enumerated from overnight cultures of serially diluted 10 mm lesioned skin punch biopsy specimen homogenized at 4°C (Pro200 Series homogenizer; Pro Scientific).

### 2.6 Pyomyositis infection model in rabbits

Two weeks after the last immunization, the IM infection was performed on the right thigh. Rabbits were anesthetized with IM ketamine (20-30 mg/kg) and xylazine (1-2 mg/kg) and general anesthesia was maintained with inhalation isoflurane (∼1.5%). Sterile ophthalmic ointment (Rugby, Livonia, MI, USA) was applied to the eyes. Metoclopramide (Teva) (0.3 mg/kg) was injected subcutaneously as a gastrointestinal promotility agent and sustained-release buprenorphine (ZooPharm) (0.2 mg/kg) was injected subcutaneously for analgesia. For infection, 6-8 ×10^9^ CFU of bioluminescent SF8300 in 100 µl of PBS was injected into the muscle located in the middle of femur with depth of 0.5 cm using a 29-gauge insulin syringe. *In vivo* BLI was performed using a Lumina III IVIS (PerkinElmer) and maximum flux (photons/s/cm2/steradian) was measured within an 8.1×6.4 cm circular region of interest using Living Image software (PerkinElmer). Thigh width was measured by Pittsburgh digital caliper (Harbor Freight Tools). Ex vivo CFU was enumerated from overnight cultures of serially diluted muscle abscess specimen homogenized at 4°C (Pro200 Series homogenizer; Pro Scientific).

### 2.7 Bacteremia infection model in mice

Lethal and sublethal doses of the three LMIC strains in BALB/c mice were first established prior to testing the efficacy of IBT-V02 in a systemic infection model. Mice were challenged with different doses of either KCO075, Pyo603 or Gaza209 via the intraperitoneal (IP) route and infective dose resulting in 60-80% mortality was selected for testing efficacy of IBT-V02 in the IP infection model. For bacteremia, six weeks after the last immunization, mice were infected intraperitoneally (IP) with a 200 µl suspension of *S. aureus* (8x10^7^ CFU/mouse of KCO075, 4x10^7^ CFU/mouse of Pyo603 or 2x10^7^ CFU/mouse of Gaza209), as modified from previously described protocols (20). The animals were monitored daily for 14 days for survival.

### 2.8 Lyophilization of IBT-V02

Differential Scanning Calorimetry was performed to determine the glass transition temperature (Tg’), collapse temperature (Tc), and impact of freezing rate for the liquid formulation (Hla_H35LH48L_, LukS_mut9_, LukF_mut1_, TBA_225_ and LukAB_mut50_ formulated at total protein 0.2mg/ml in 20mM histidine-borate, 1.2mM phosphate buffer with 8% sucrose and 0.02% Tween 80). The average values for Tg’ and Tc obtained from several experiments were used to design the lyophilization process, with primary drying set at -40°C (5°C below Tc) and annealing at -25°C (at least 5°C above the Tg’). Traditional lyophilization was accomplished by freezing the liquid vaccine and then reducing the surrounding pressure and applying enough heat to allow the frozen water in the material to sublime. During primary drying, the pressure in the surrounding environment was lowered to facilitate sublimation. In secondary drying the remaining water molecules were removed, bringing the lyophilized vaccine down to a final moisture content of between 3 and 5%. The result of the process was a “cake” of powder that could be reconstituted in water at time of use. In addition to traditional lyophilization, a spray freeze-drying process was also incorporated where a carefully formulated liquid solution was atomized into specifically sized spherical droplets (determined by spray nozzle orifice) and immediately frozen, locking in the size and shape of each individual particle. The particles were collected and dried in bulk in a standard lyophilization chamber. Both traditional lyophilization and spray freeze-drying processes were run at Engineered BioPharmaceuticals to produce small-scale batches to determine feasibility followed by production of large-scale batches. For long-term storage studies, lyophilized IBT-V02 formulations were kept at -20°C and 30°C for 1, 3, 6, 9, or 12 months.

### 2.9 Biochemical/biophysical characterization of lyophilized vaccine by BCA, SDS-PAGE, Western blot, and size exclusion high performance liquid chromatography

**Figure 1:**
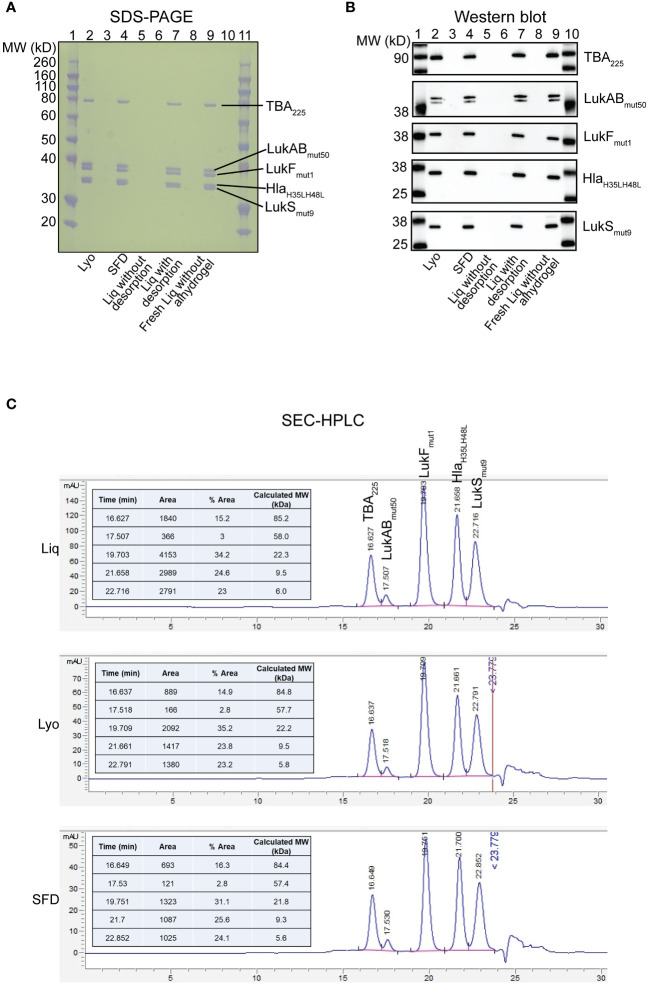
Biochemical characterization of IBT-V02 components in Lyo, SFD and Liq vaccine formulations. **(A)** SDS-PAGE and **(B)** Western Blot analysis of Lyo (Lane 2), SFD (Lane 4), Liq without desorption (Lane 6), Liq with desorption (Lane 7) and freshly prepared Liq without alhydrogel (Lane 9). Samples were loaded at 1µg total protein for SDS-PAGE and 100ng for all individual proteins in Western Blot, except LukAB_mut50_ which was loaded at 250ng. **(C)** SEC-HPLC of Liq (Top), Lyo (Middle) and SFD (Bottom).

The lyophilized and spray freeze-dried (SFD) IBT-V02 were characterized by several biophysical/biochemical assays. Briefly, Bicinchoninic acid protein assay (BCA) was used to confirm the protein concentrations after reconstitution of lyophilized/SFD IBT-V02 in sterile water. SDS-PAGE was run at protein loads as indicated in **Figure 1A** using a 12% Bis-Tris gel. For WB, primary antibodies which were antigen-specific and goat anti-rabbit IgG alkaline phosphatase conjugate (Bio-Rad) secondary antibody (1:3000, v/v) were used. For SEC-HPLC, 50µg of Lyo, SFD, or liquid IBT-V02 were injected in an Agilent Technologies 1260 Infinity Series instrument using an AdvanceBio SEC 300Å 7.8x300 mm LC column with a mobile phase of 50 mM sodium phosphate buffer + 150 mM NaCl, pH 7.0 running at a flow rate of 0.5 mL/min. The chromatogram generated by the Agilent OpenLabs software plots absorbance at 280 nm as a function of retention time. All analysis of the peaks was performed by the auto-integrate function in the OpenLabs software.

### 2.10 Preparation of supernatants

Bacterial cells were streaked on sheep blood agar plates and incubated at 37°C overnight for 16 hours. Next, 7ml of BHI broth were inoculated with 4-6 colonies from each plate, incubated at 37°C at 230rpm for 16 hours, and OD_600_ was measured. Cells were centrifuged at 3500rpm, and supernatant was collected. The volume of supernatants among different cultures was normalized to a bacterial OD_600 = _6.0 with BHI broth. The normalized supernatants were then sterile-filtered and stored at -80°C.

### 2.11 Determination of hemolytic activities on blood agar plates

Qualitative α-, β- and δ-hemolysin production was evaluated on sheep blood agar plates, as previously described (25). Briefly, bacterial strains were streaked perpendicular to a β-hemolysin producing tester strain, RN4220. The inoculated plate was incubated at 37°C for 16-18 hours. Results were determined as follows: δ-hemolysin and β-hemolysin act synergistically, which produces a clearing zone at their intersection. α-hemolysin, and β-hemolysin inhibit each other, which results in a V-shaped zone where they intersect. When a strain is β-hemolytic, the clearing blends in with the tester strain. When a strain is both α- and δ-hemolytic, there is a stronger clearing on the characteristic V-shaped zone.

### 2.12 HL-60 cytotoxicity assay

Induced HL-60 cells were harvested by centrifugation at 1200 rpm for 10 minutes at 20°C (12, 20). Cells were washed and resuspended with phenol red-free RPMI 1640 (Gibco) supplemented with 2% FBS to a final concentration of 5 x 10^6^ cells/ml. Supernatants were serially diluted 2-fold across 96-well plates (50 µl/well) and 100µl of cells were added to each well. Plates were incubated at 37°C, 5% CO_2,_ 95% humidity for 3 hours. After 3 hours, 50 μl of reconstituted CellTiter Glo reagent (Promega) was added to each well. The plate was shaken on an orbital shaker for 10-15 min at room temperature followed by measurement of luminescence (emission at 560 nm) using Cytation 5 imaging reader (Biotek) and Gen5 2.09 software to determine cell viability.

### 2.13 Toxin neutralization assay in HL-60 cells

Polyclonal rabbit antibody against IBT-V02 was diluted 2-fold in phenol-red RPMI across 96-well plate starting at 500µg/ml (25 µl/well) (12, 20). Equal volumes of supernatants were added at determined concentrations. 100µl of prepared induced HL-60 cells were added to each well and incubated at 37°C, 5% CO_2,_ 95% humidity. Cell viability was determined with CellTiter Glo reagent, as described above.

### 2.14 Toxicity in rabbit red blood cells

10ml PBS was added to 5ml of rabbit blood (Colorado Springs) and centrifuged at 1500rpm for 10 minutes at 20°C (12, 20). Supernatant was discarded and pellet was washed with 13ml PBS. The final pellet was weighed and diluted in PBS to 8% w/v for rabbit blood.

Bacterial supernatants were serially diluted 2-fold in PBS across 96-well plates (100 µl/well). 100 µl of prepared red blood cells were added to each well. The plate was incubated at 37°C for 30 minutes and then centrifuged at 3500rpm for 3 minutes at 20°C. Supernatants were transferred to 96-well plates compatible with plate reader. Toxicity was determined by measuring absorbance at 416nm using Spectramax 190 plate reader (Molecular Devices) and Softmax 5.4.5 software.

### 2.15 Toxin neutralization assay in rabbit red blood cells

Polyclonal rabbit antibody against IBT-V02 was diluted 2-fold in PBS across 96-well plate starting at 500µg/ml (50 µl/well) (12, 20). Equal volumes of supernatants were added at determined concentrations. 100µl of prepared rabbit red blood cells were added to each well and incubated at 37°C, 5% CO_2,_ 95% humidity. Neutralization was determined by measuring absorbance at 416nm using Spectramax 190 plate reader (Molecular Devices) and Softmax 5.4.5 software.

### 2.16 Western blots for bacterial supernatants

Supernatant dilutions consisted of 75% supernatant and 25% 4X Laemli’s reducing buffer (Boston BioProducts). Dilutions were denatured at 70°C for 10 minutes or at 100°C for 5 minutes. Gels were run for 35 minutes at 165V. Blots were transferred using a standard 7-minute procedure using iBlot 2 (Thermo Fisher Scientific).

Blots were blocked with StartingBlock (TBS) blocking buffer (Thermo Fisher Scientific) for 10 minutes and incubated in primary antibodies specific to different Staphylococcal toxins: Hla (Cat# Hla-ID-P), LukS-PV(Cat# LukS-ID-P), LukAB (cat# LukAB-ID-P), LukF-PV (Cat# LukF-IP-T01), TSST-1 (cat# TSST-1-ID-P-02), SEB (Cat-SEB-ID-P), and SEA (cat# SEA_ID-02) (all generated by IBT Bioservices) for 18 +/- 2 hours (26). This was followed by 2 rapid washes and a third 5-minute wash in 1X TBS-T (Thermo Fisher Scientific). Secondary antibody was diluted at 1:3000, and blots were incubated in it for 50-60 minutes. The blots were finally washed three times as described earlier. Blots incubated in AP- or HRP-conjugated secondary antibodies were washed in 1X TBS for 5 minutes. When using AP-conjugated secondary antibody (Bio-Rad), blots were developed using AP detection buffer (Bio-Rad) and imaged with a camera (Azure system). The blots incubated in HRP-conjugated secondary antibodies (KPL) were developed with ECL reagent (Azure) and imaged with Azure 600 imager.

### 2.17 Genotyping of isolates from low to middle income countries

QiAmp DNA Mini Kit (Qiagen, Cat# 51306) was used to extract and purify DNA from the bacterial isolates. A small number of pure colonies (5, 6) bacteria were mixed with 200 µL of purified water as a starting material. The DNA was extracted by following the manufacture’s protocol. The following are the PCR reagents and conditions used for PCRs. PCR Reagents: 15µl of HotStarTaq ™ Master Mix, 1µl of Forward primer (20 pmol/µl), 1µl of Reverse primer (20 pmol/µl), 3µl of extracted DNA for the template, 0.6 µl of 50 mM Mg2+, 9.4 µl of dH2O PCR Conditions: i.) Initial denaturation 95°C for 15 minutes; ii.) 35 cycles of Denaturation 94°C for 30 seconds, Annealing 55°C for 30seconds, and Elongation 72°C for 45 seconds; iii.) Final Elongation 72°C for 10 minutes; and iv.) Hold at 4°C. Genotyping primers are listed in **Supplementary Table 1**. Genotyping data were generated at Marshfield clinic under the supervision of Dr. Shukla.

### 2.18 Serum total antibody titers

A multiplex assay to detect serum IgG titers to *S. aureus* antigens has been previously developed at IBT using the Luminexⓒ xMAP^®^ technology (20). Briefly, IBT-V02 target antigens Hla, LukS-PV, LukF-PV, LukAB, SEA, SEB, and TSST-1 were coupled to carboxylated MagPlex microsphere beads with distinct spectral regions via a carbodiimide reaction. Antigen-coupled beads were incubated with serum samples (mouse or rabbit) at a starting dilution of 1:40 in a two-fold 8-point dilution series at room temperature (RT) for 2 hours. Samples were washed and incubated with a PE-conjugated goat anti-mouse or donkey anti-rabbit IgG Antibody (Biolegend, San Diego, CA.) for one hour at RT. The samples were washed and acquired using a Luminex200. Data were analyzed using a 4-parameter (4PL) curve fit in XLFit (Microsoft). IgG titers were expressed as the effective dilution at the point of the 4PL curve where 50% (ED_50_) of antigen was detected by toxin-specific antibodies present in the serum sample.

### 2.19 Serum total neutralizing titers

Hla TNAs were performed as previously described (27). In brief, 4% rabbit red blood cells (RRBCs) were co-cultured with wild-type Hla ± serially diluted serum samples. Cells were centrifuged after 30 min at 37°C, and absorbance determined at OD416 nm. PVL and LukAB TNAs were performed with human promyelocytic leukemia (HL-60) cells as previously described (28). In brief, differentiated HL-60 cells were incubated with either PVL or LukAB ± serially diluted serum samples for 3 hours at 37°C, and CellTiter Glo reagent (Promega) was added to the culture to measure cell viability. Superantigen (SAg) TNAs were performed with human PBMCs isolated from Leukopaks (StemExpress). Cells were co-cultured with SEA, SEB, or TSST-1 in the presence or absence of serially diluted serum samples for 48 hours; supernatants were collected, and IFNγ was measured in the supernatants as a readout of superantigenicity as we previously described (29). Data was analyzed using a 4-parameter (4PL) curve fit with XLFit (Microsoft). Toxin neutralizing activity was defined as the effective dilution of sera at the point of the 4PL curve at which 50% of toxin activity was neutralized (ND_50_).

### 2.20 Statistical analyses

Data between more than two groups for longitudinal comparisons (*in vivo* BLI, lesion size) were compared using a two-way analysis of variance (ANOVA) multiple comparisons test with Dunnett correction. For single time point comparisons (abscess weight and ex vivo CFU), groups were compared using a one-way ANOVA-Kruskal-Wallis multiple comparisons test with Dunn’s correction, and survival rates were compared by log rank (Mantel-Cox) test, as indicated in the figure legends. All statistical analyses were calculated with Prism software (GraphPad 10.3 Software, La Jolla, California). Data are presented as mean or geometric mean (for *ex vivo* CFU data) ± standard error of the mean (SEM) and values of *P <*0.05 were considered statistically significant.

## 3 Results

### 3.1 Solid and liquid IBT-V02 formulations have comparable protein characterization

To determine the biochemical characterization of the IBT-V02 toxoid vaccine components in our solid lyophilized (Lyo) and spray freeze-dried (SFD) formulations compared to our liquid (Liq) formulation, we performed SDS-PAGE, Western Blot, and SEC-HPLC analyses (**Figure 1**). First, we examined the formulations by SDS-PAGE and found that overall protein integrity was intact in the Lyo (lane 2), SFD (lane 4), Liq without desorption (lane 6), Liq with desorption (Lane 7), and freshly prepared Liq without alhydrogel (Lane 9) formulations (**Figure 1A**). Next, we performed a Western blot on vaccine toxoid components from the same conditions described above and verified that neutralizing antibody binding specificity was maintained in all the formulations tested (**Figure 1B**). Finally, we performed SEC-HPLC, which confirmed our SDS-PAGE and Western blot results that protein constitution of the IBT-V02 vaccine toxoid components is comparable between the Liq, Lyo, and SFD formulations tested (**Figure 1C**).

### 3.2 Solid IBT-V02 formulations maintain long-term stability at room temperature storage

Given the difficulty in maintaining cold chain storage in LMICs (30), we next examined the stability of the Lyo and SFD IBT-V02 formulations at varying storage times and temperatures, and compared the stability to the freshly prepared Liq formulation. First, we tested the storage stability of the IBT-V02 formulations at -20°C and 30°C for 1- and 3-month durations. Excitingly, we found that vaccine components of both Lyo and SFD formulations had comparable stability to the freshly prepared Liq formulation by SDS-PAGE (**Supplementary Figures 1A, C**) and SEC-HPLC (**Supplementary Figures 1B, D**) analyses. We next tested the storage stability of the Lyo formulation at -20°C and 30°C for 6-, 9-, and 12-month durations. Similar to our findings at 1 and 3 months, the Lyo formulation had comparable biochemical stability as freshly prepared Liq IBT-V02 formulation at 6, 9, and 12 months of storage (**Supplementary Figure 2**). Collectively, our findings suggested that the Lyo and SFD IBT-V02 formulations effectively maintain long-term stability at room temperature storage.

### 3.3 Comparative immunogenicity of solid and liquid IBT-V02 formulations in vaccinated mice

**Figure 2:**
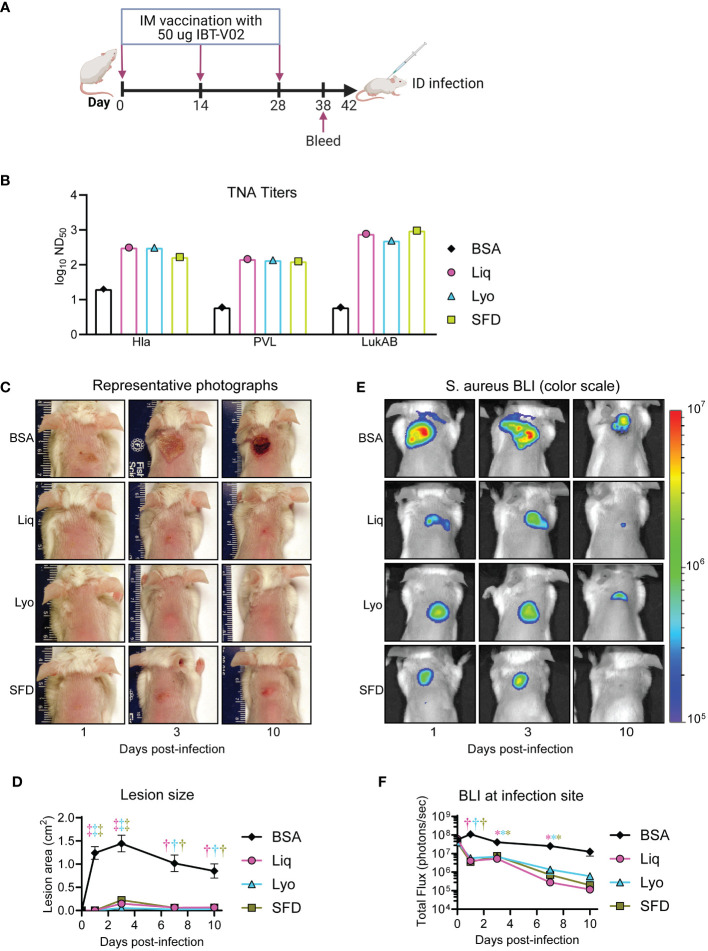
Comparative immunogenicity and efficacy in a murine intradermal infection model. Mice (n=10 mice per group) were immunized with 50 µg of either Liq, Lyo, or SFD formulations of IBT-V02 or 50 µg of BSA in 200µg of Alhydrogel (Al(OH)_3_) on days 0, 14 and 28. On day 38, sera were collected to assess toxin neutralizing antibody titers in cell-based assays. On day 42, MRSA (8×10^6^ CFU of the bioluminescent MRSA strain SF8300) skin infections were performed on immunized mice and bacterial burden and lesion size monitored until the experiment was arbitrarily ended after 10 days. **(A)** Model timeline. **(B)** Toxin neutralizing antibody titers. **(C)** Representative photographs of skin lesion. **(D)** Mean total lesion size (cm^2^) ± SEM. **(E)** Representative *in vivo* BLI (photons/s) (log_10_ scale). **(F)** Mean total flux (photons/s) ± SEM. *P < 0.05, ^†^P < 0.01, ^‡^P < 0.001, as calculated by a two-way analysis of variance (ANOVA) Dunnett’s multiple comparisons test **(D, F)**. Results are combined from two independent experiments.

After testing the long-term biochemical stability of the solid formulations, we next set out to compare the immunogenicity of the solid and liquid IBT-V02 formulations in vaccinated mice. To this end, mice were immunized with 50 µg of either Liq, Lyo, or SFD formulations in 200µg of Alhydrogel (Al(OH)_3_) on days 0, 14 and 28 (**Figure 2A**). On day 38, 10 days after the final immunization, sera were collected to assess toxin binding IgG titers by Luminex, and toxin neutralizing antibody titers in cell-based assays, as previously described (12). We found that all three formulations of IBT-V02 induced similar levels of toxin neutralizing capacity when tested against target antigens, which were increased over BSA-controls (**Figure 2B**). Importantly, IBT-V02 immunogenicity was maintained in the Lyo formulation after long-term storage (6 months) at -20C and 30C compared to fresh preparations (**Supplementary Figure 3**), indicating the viability of the solid formulations as an alternative to IBT-V02 vaccination with the liquid formulation in mice.

### 3.4 Comparative efficacy of solid and liquid IBT-V02 formulations in a murine intradermal infection model

We next compared the protective efficacy of Liq, Lyo, and SFD IBT-V02 formulations compared to BSA controls in a mouse model of MRSA skin infection, whereby mice were injected i.d. with 8×10^6^ CFU of the bioluminescent MRSA strain, SF8300, and bacterial burden and lesion size monitored until the experiment was arbitrarily ended after 10 days. We used *in vivo* bioluminescence imaging (BLI) to non-invasively monitor bacterial burden, which we have previously shown to correlate with *ex vivo* CFUs (R2 = 0.9996) (31). We discovered that Liq, Lyo, and SFD vaccinated mice had significantly reduced lesion sizes (**Figures 2C, D**) and bacterial burdens (**Figures 2E, F**) compared to BSA-treated controls. Taken together, our findings indicated that Liq, Lyo, and SFD IBT-V02 formulations had significant efficacy against MRSA skin infections in mice.

### 3.5 Comparative immunogenicity of solid and liquid IBT-V02 formulations in vaccinated rabbits

**Figure 3:**
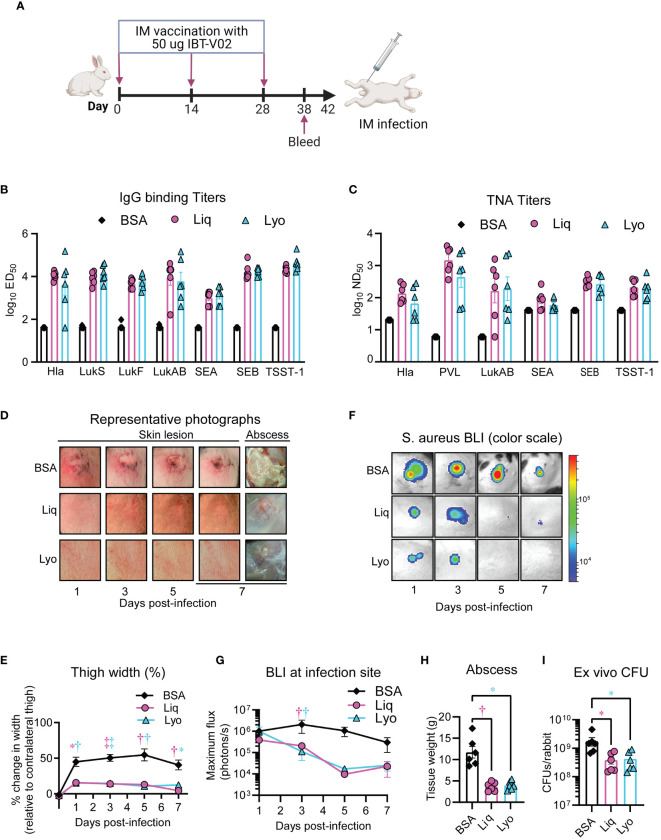
Comparative immunogenicity and efficacy in rabbit pyomyositis. Rabbits (n=6 rabbits per group) were immunized with 50 µg of either Liq or Lyo formulations of IBT-V02 or 50 µg of BSA in 200µg of Alhydrogel (Al(OH)_3_) on days 0, 14 and 28. On day 38, sera were collected to assess toxin binding IgG titers by Luminex, and toxin neutralizing antibody titers in cell-based assays. On day 42, MRSA (6-8 ×10^9^ CFU of the bioluminescent MRSA strain SF8300) pyomyositis infections were performed on immunized rabbits and skin lesions and thigh width monitored until the experiment was arbitrarily ended after 7 days, when abscesses were imaged, weighted, and harvested for ex vivo CFUs. **(A)** Model timeline. **(B)** Toxin-binding IgG titers. **(C)** Toxin neutralizing antibody titers. **(D)** Representative photographs of skin lesion and abscess. **(E)** Mean thigh width change (% of contralateral thigh) ± SEM. **(F)** Representative *in vivo* BLI images. **(G)** Mean *in vivo* BLI signals quantified as maximum flux (photons/s/cm^2^/steradian) ± SEM. **(H)** Mean tissue weight of abscess ± SEM. **(I)** Geometric mean of ex vivo CFUs ± SD. *P < 0.05, ^†^ *P* < 0.01, ^‡^ *P* < 0.001, as calculated by a two-way analysis of variance (ANOVA) Dunnett’s multiple comparisons test **(E, G)** or Kruskal-Wallis Dunn’s multiple comparisons test **(H, I)**. Results are combined from two independent experiments.

Since *S. aureus* pore-forming toxins that are neutralized by the IBT-V02 vaccine have higher affinity to rabbit immune cells than mice (32), we next wanted to compare the immunogenicity of the dry and liquid IBT-V02 formulations in vaccinated rabbits. Given the similar outcomes between SFD and Lyo formulations in the murine intradermal infection model and the successful use of lyophilization in commercialized vaccines in LMICs (e.g., MenAfriVac) (18, 33), we herein focused our comparative studies on the Lyo IBT-V02 formulation. To this end, rabbits were immunized with 50 µg of either Liq or Lyo formulations in 200µg of Alhydrogel (Al(OH)_3_) on days 0, 14 and 28 (**Figure 3A**). On day 38, 10 days after the final immunization, sera were collected to assess toxin binding IgG titers by Luminex, and toxin neutralizing antibody titers in cell-based assays, as previously described (12). We found that the Liq and Lyo IBT-V02 formulations had similar trends in the increased levels of toxin-binding IgG titers (**Figure 3B**) and toxin-neutralizing antibodies (**Figure 3C**) compared to BSA-treated rabbits.

### 3.6 Comparative efficacy of solid and liquid IBT-V02 formulations in a novel rabbit pyomyositis infection model

Pyomyositis is a major health burden in LMICs and increasing in prevalence in high-income countries (3), with *S. aureus* as the predominant etiological agent (2). Therefore, we developed a rabbit pyomyositis infection model to compare the protective efficacy of Lyo and liquid IBT-V02 formulations whereby rabbit thighs were injected intra-muscularly (IM) with 6-8 ×10^9^ CFU of *S. aureus* strain SF8300 and skin lesions and thigh width monitored until the experiment was arbitrarily ended after 7 days, when abscesses were imaged, weighed, and harvested for *ex vivo* CFUs. Lyo and Liq treated rabbits had marked reductions in gross skin lesion and abscess sizes (**Figure 3D**) and thigh widths (**Figure 3E**) compared to BSA-treated rabbits. Furthermore, there was a decrease in BLI in vaccinated rabbits compared to BSA-treated controls (**Figures 3F, G**), which correlated with significant reductions in abscess weights (**Figure 3H**) and *ex vivo* CFUs (**Figure 3I**). Thus, our data suggested that Lyo and Liq IBT-V02 formulations had comparable and significant efficacy in a novel rabbit pyomyositis infection model.

### 3.7 LMIC isolate down-selection strategy via *in vitro* toxin characterization

**Figure 4:**
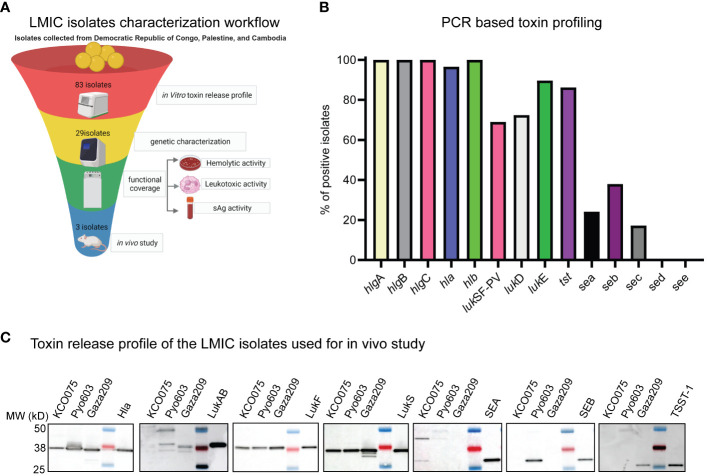
Characterization of LMIC isolates *in vitro*. **(A)** LMIC isolates characterization workflow. **(B)** PCR based toxin profiling. **(C)** Toxin release profile of the LMIC isolates used for *in vivo* study.

Since we only tested efficacy of IBT-V02 against *S. aureus* isolates from the U.S. in our preclinical infection models (12), we wanted to confirm the efficacy of the Lyo and Liq IBT-V02 formulations against *S. aureus* isolates from LMICs. Thus, we acquired a total of 83 clinical isolates of *S. aureus* from pyomyositis patients in the Democratic Republic of Congo, Palestine, and Cambodia. Since testing 83 clinical isolates *in vivo* is cost-prohibitive, we created a rational multi-dimensional down-selection strategy to screen isolates that exhibited broad toxin production for testing the efficacy of our vaccine formulations (**Figure 4A**). First, we measured toxin release via Western blot to down-select to 29 clinical isolates.

Next, we performed a toxin profile PCR assay to identify isolates that were positive for toxins neutralized by IBT-V02 (**Figure 4B**; **Supplementary Table 2**). We discovered that 100% of the isolates had the hla, hlgA, B, C, and hlb genes, 90% of the isolates had the LukE and tst, 60-70% of isolates had the PVL and LukD genes, and less than 40% of isolates had the Sea, Seb, and Sec genes (**Figure 4B**). However, we did not detect Sed and See in any of the isolates (**Figure 4B**). Out of the 29 isolates analyzed, 25 of them had more than 4 leukotoxins genes other than PVL. Furthermore, 22 of the isolates had 1-2 superantigen (SAg) genes (**Supplementary Table 2**). Only one isolate had 4 SAg genes, 4 isolates had 3, and 3 isolates did not have any SAg genes. Furthermore, we typed these isolates using 18 different genes as typing markers. Among them, 14 were toxin genes, and 4 (SPA type, ST, CC, and SCCmec) were the established epidemiological typing genes. Out of 29 isolates, 15 were MSSA isolates, and 14 were MRSA isolates. Among MRSA isolates, SCCmec type IVA was the most predominant (12 out of 14), and 2 were SCCmec type III. The most common CC types were CC22 and CC121 whereas the SPA types were highly diverse.

To confirm toxin protein production by the down-selected isolates, we performed *in vitro* hemolytic and leukocytic activity assays using rabbit red blood cells and neutrophils, respectively. From our multi-dimensional screen, we identified 3 clinical isolates to use in *in vivo* studies, one from each of the Democratic Republic of Congo, Palestine, and Cambodia that expressed the lytic toxins and at least one superantigen. These strains were named KCO075, Pyo603, and Gaza209, which in addition to the lytic toxins produced SEA, SEB, and TSST-1, respectively (**Figure 4C**).

### 3.8 *In vitro* neutralization of culture supernatants by IBT-V02 pAbs

To assess the potential efficacy of IBT-V02 against the down-selected isolates from LMICs, we explored the neutralizing potential via *in vitro* hemolytic and leukocytic activity assays. The toxicity of each bacterial supernatant (SUP) was first determined in rabbit red blood cells and HL-60 cells (**Supplementary Figure 4**). SUPs were prepared as described previously (20). Starting at neat, cells were incubated with 11-point dilutions of bacterial SUPs and toxicity was measured. Data were analyzed using a 4-parameter (4PL) curve fit in XLFit (Microsoft) and the effective dilution at the point of the 4PL curve where ED_85_ toxicity occurred was calculated. Cells were then incubated with the ED_85_ dilution of SUPs in the presence or absence of decreasing concentrations of IBT-V02 rabbit pAbs. The neutralizing concentration (NC) at the point of the 4PL curve where 50% neutralization of SUP toxicity occurred (NC_50_) was then calculated. As shown in **Supplementary Figure 4**, pAbs raised against IBT-V02 showed neutralizing capacity towards the SUPs of all three LMIC isolates at different concentrations. For RRBCs the NC_50_ ranged from 19.3-26.4µg/ml and for HL60 cells from 20.5-60.9µg/ml.

### 3.9 Protective efficacy of IBT-V02 formulations against LMIC isolates in a bacteremia infection model in mice

**Figure 5:**
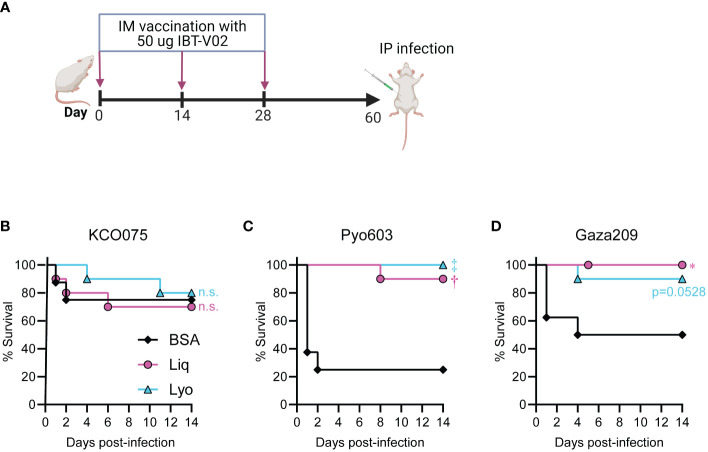
Protective efficacy of IBT-V02 against LMIC isolates in a mouse bacteremia model. Mice (n=10 mice per group) were immunized with 50 µg of either Liq or Lyo formulations of IBT-V02 or 50 µg of BSA in 200µg of Alhydrogel (Al(OH)_3_) on days 0, 14 and 28. Six weeks post-immunization, the LMIC bacteremia infection was performed on immunized mice. **(A)** Model timeline. **(B)** Survival (%) of mice infected with 8x10^7^ CFU of KCO075. **(C)** Survival (%) of mice infected with 4x10^7^ CFU of Pyo603. **(D)** Survival (%) of mice infected with 2x10^7^ CFU of Gaza209. *P < 0.05, ^†^ *P* < 0.01, ^‡^ *P* < 0.001, BSA versus Liq or Lyo as calculated by log rank (Mantel-Cox) test. n.s., not significant.

*S. aureus* bacteremia has a 20-30% mortality rate in high-income countries (34, 35), which is elevated to over 50% in LMICs (36, 37). Thus, to evaluate the *in vivo* efficacy of our IBT-V02 formulations against LMIC isolates we immunized mice with 50 µg of either Liq or Lyo formulations in 200µg of Alhydrogel (Al(OH)_3_) on days 0, 14 and 28 (**Figure 5A**). On day 60, we performed a *S. aureus* bacteremia infection model in mice whereby KCO075, Pyo603, and Gaza209 isolates were injected intraperitoneally into BSA, Liq, or Lyo treated mice and survival monitored over time until day 14 when the experiments were arbitrarily ended. Mice infected with the KCO075 isolate had comparably low reductions in survival rates between all groups tested (**Figure 5B**). However, mice treated with the Liq and Lyo IBT-V02 formulations had significant increases in survival compared to BSA-treated mice when infected with the Pyo603 isolate (**Figure 5C**). Lastly, we found a similar trend in mice infected with the Gaza209 isolate, whereby Liq and Lyo vaccinated mice had markedly increased survival rates compared to BSA control mice (**Figure 5D**). Collectively, our data suggested that Liq and Lyo IBT-V02 formulations have protective efficacy against LMIC isolates in a *S. aureus* bacteremia infection model.

### 3.10 Protective efficacy of IBT-V02 formulations against LMIC isolates in a murine intradermal infection model

**Figure 6:**
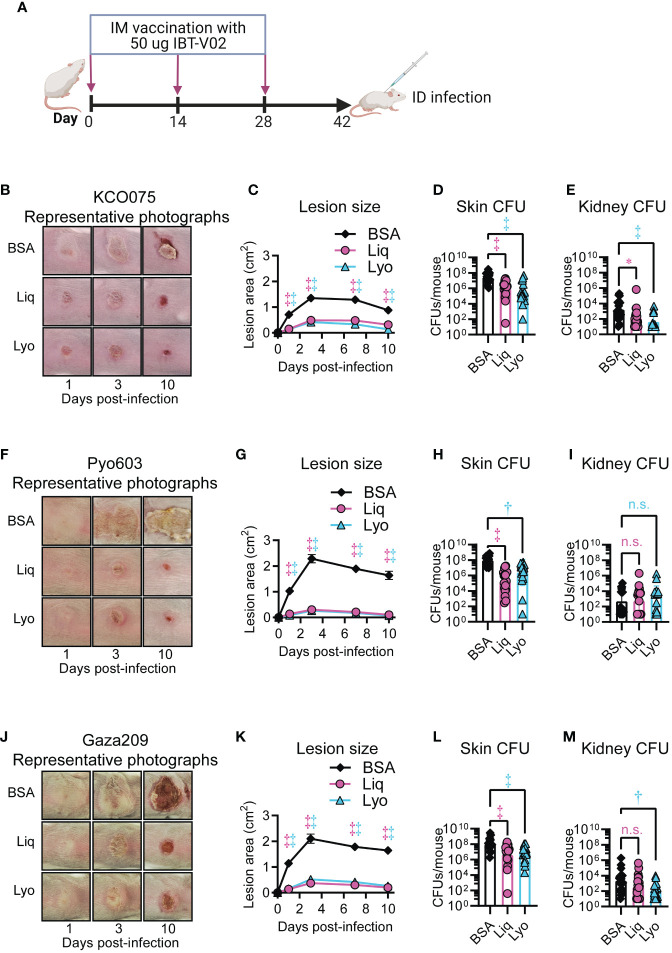
Protective efficacy of IBT-V02 against LMIC isolates in a mouse intradermal infection model. Mice (n=10 mice per group) were immunized with 50 µg of either Liq or Lyo formulations of IBT-V02 or 50 µg of BSA in 200µg of Alhydrogel (Al(OH)_3_) on days 0, 14 and 28. On day 42, intradermal infections with 1x10^9^ CFU of KCO075 **(B–E)**, 3x10^8^ CFU of Pyo603 **(F–I)**, and 2x10^8^ CFU of Gaza209 **(J–M)** were performed on immunized mice and skin lesions monitored until the experiment was arbitrarily ended after 10 days, when skin and kidney were harvested for ex vivo CFUs. **(A)** Model timeline. **(B, F, J)** Representative photographs of skin lesion. **(C, G, K)** Mean total lesion size (cm^2^) ± SEM. **(D, H, L)** Geometric mean of skin lesion ex vivo CFUs ± SD. **(E, I, M)** Geometric mean of kidney ex vivo CFUs ± SD. *P < 0.05, ^†^ *P* < 0.01, ^‡^ *P* < 0.001, as calculated by a two-way analysis of variance (ANOVA) Dunnett’s multiple comparisons test **(C, G, K)** or Kruskal-Wallis Dunn’s multiple comparisons test **(D, E, H, I, L, M)**. Results are combined from two independent experiments. n.s., not significant.

We next tested the efficacy of the Liq and Lyo IBT-V02 formulations against LMIC isolates KCO075, Pyo603, and Gaza 209 in the mouse intradermal infection model as described above. Mice were immunized with 50 µg of either Liq or Lyo formulations in 200µg of Alhydrogel (Al(OH)_3_) on days 0, 14 and 28 (**Figure 6A**). Mice infected with *S. aureus* isolate KCO075 and vaccinated with Liq or Lyo formulations had significant reductions in lesions sizes compared to BSA-treated mice (**Figures 6B, C**). These results corresponded with markedly reduced *ex vivo* CFUs from the skin and kidneys of Liq and Lyo treated mice compared with BSA-treated mice (**Figures 6D, E**). Similar to mice infected with KCO075, Liq and Lyo treated mice infected with Pyo603 had markedly reduced lesion sizes and *ex vivo* CFUs from skin compared to BSA controls (**Figures 6F–H**). However, Pyo603-infected mice did not have differences in *ex vivo* CFUs from kidneys between all the groups tested (**Figure 6I**). Lastly, Liq and Lyo treated mice infected with Gaza209 had significantly dampened lesion sizes and *ex vivo* skin CFUs compared to BSA-treated mice (**Figures 6J–L**), with *ex vivo* kidney CFUs significantly lessened only in the Lyo treated mice (**Figure 6M**). Taken together, Liq and Lyo IBT-V02 formulations were effective against *S. aureus* isolates from LMICs in a mouse intradermal infection model.

## 4 Discussion

The emergence of antibiotic-resistant *S. aureus* is a global health burden (4), which disproportionately affects LMICs. Although we have previously shown efficacy of IBT-V02 in liquid formulations against *S. aureus* isolates from the industrialized world (12), whether protection is maintained in dry formulations against *S. aureus* isolates from LMICs has not been established. Therefore, we developed and tested lyophilized and spray freeze-dried formulations of IBT-V02 compared to the liquid formulation for protein stability, immunogenicity, and *in vivo* efficacy in multiple preclinical models against *S. aureus* isolates from the U.S. and LMICs. Our investigation revealed that the dry formulations of IBT-V02 are comparable to the liquid formulation in protein stability during long-term storage at room temperature, immunogenicity against *S. aureus* toxins, and protective efficacy against *S. aureus* isolates from LMICs in murine and rabbit infection models. These results provide several important insights into the protective efficacy of IBT-V02 during *S. aureus* infections.

First, we found that the lyophilized and freeze-dried IBT-V02 formulations had preserved protein stability after reconstitution, even after long-term storage at room temperature. These findings recapitulated the prevailing literature that dry vaccine formulations have decreased sensitivity to temperature-induced degradation (38). Given the difficulty in maintaining cold chain storage in LMICs, our results suggested that dry formulations of IBT-V02 have potential to be distributed in warm and tropical climates without reductions in vaccine component integrity. This was further corroborated in our immunogenicity studies, which found that mice and rabbits vaccinated with the liquid and dry formulations had comparable induction of anti-toxin neutralizing titers compared to BSA-treated controls. Our findings are similar to the modified vaccinia Ankara vaccine for smallpox, whereby a lyophilized vaccine formulation had comparable immunogenicity as a liquid formulation (39). Collectively, our results indicated that lyophilized and spray freeze-dried IBT-V02 are viable formulations for maintaining vaccine immunogenicity during long-term storage at room temperature.

We also discovered that the dry and liquid IBT-V02 formulations had comparable protective efficacy against a *S. aureus* USA300 strain in an intradermal infection model in mice, which replicates our previous findings using the liquid IBT-V02 formulation against *S. aureus* isolates from the U.S (12). Moreover, the lyophilized and liquid IBT-V02 formulations had comparable protective efficacy in a pyomyositis infection model in rabbits. This is important, as *S. aureus* pore-forming toxin leukocytic activity in rabbits is more comparable to humans than mice (32), enhancing the relevancy of our results. However, studies to determine whether IBT-V02 sustains protective efficacy during *S. aureus* infections in humans are warranted and will be the focus of future clinical trials.

Our findings revealed that dry and liquid IBT-V02 formulations promoted survival during bacteremia infections with multiple isolates of *S. aureus* from LMICs. Although we did not find a benefit for IBT-V02 in mice infected with LMIC isolate KCO075, this may be due to reduced toxin expression of the KCO075 isolate, which had lower RRBC and HL-60 toxicity compared to Pyo603 and Gaza209 strains (**Supplementary Figure 4**). Similar to our current and published results with U.S. isolates of *S. aureus* (12), dry and liquid IBT-V02 formulations had protective efficacy against multiple isolates from LMICs in an intradermal infection model in mice. However, IBT-V02 vaccination did not reduce the bacterial dissemination to the kidneys of Pyo603-infected mice, despite markedly decreased lesion sizes and bacterial burdens in the skin. A possible explanation is that the Pyo603 isolate is a ST121 subset strain, which have been shown to require prolonged hospitalizations and antimicrobial therapy in humans (40), and have heightened virulence in rabbits (41). Understanding the strain level variation between LMIC isolates and vaccination outcomes will be interrogated in our future studies.

There were several limitations to our study. First, we only tested the immunogenicity and protective efficacy of IBT-V02 using the IM/subcutaneous delivery method. To improve the potential vaccination rate of IBT-V02 in LMICs, we will examine vaccination delivery methods that have reduced training requirements (e.g., oral, nasal, dermal) compared to IM delivery in our future work (42, 43). Furthermore, we did not determine whether prior *S. aureus* exposure caused decreased protective efficacy of lyophilized IBT-V02, which has been reported to influence vaccine responses in other *S. aureus* infection models (44). This will be investigated in our subsequent studies.

In conclusion, dry and liquid formulations of IBT-V02 had comparable stability, immunogenicity, and protective efficacy in preclinical infection models with *S. aureus* isolates from the U.S. Importantly, dry and liquid formulations of IBT-V02 mediated protection against LMIC strains of *S. aureus*. Collectively, these findings indicated that vaccination with IBT-V02 dry formulations is a viable strategy to overcome cold chain storage limitations to protect against *S. aureus* isolates from LMICs.

## Data availability statement

The raw data supporting the conclusions of this article will be made available by the authors, without undue reservation.

## Ethics statement

All animals were maintained under the same specific pathogen-free conditions, with air-isolated cages at an American Association for the Accreditation of Laboratory Animal Care (AAALAC)-accredited animal facility at Johns Hopkins University and Integrated BioTherapeutics. They were handled according to procedures described in the Guide for the Care and Use of Laboratory Animals as well as Johns Hopkins University’s policies and procedures as outlined in the Johns Hopkins University Animal Care and Use Training Manual. Studies at JHU were approved by the Johns Hopkins Animal Care and Use Committee (Protocol #: MO21M378 for mice, Protocol#: RB22M400 for rabbit). Mouse studies at IBT were approved by institutional animal care and use committees (IACUC) (Immunogenicity Protocol# AP160805 and Efficacy against bacterial challenge Protocol#: AP-161007). The study was conducted in accordance with the local legislation and institutional requirements.

## Author contributions

YW: Data curation, Formal analysis, Investigation, Methodology, Visualization, Writing – original draft, Writing – review & editing. IM: Data curation, Formal analysis, Investigation, Visualization, Writing – original draft, Writing – review & editing. AV: Formal analysis, Investigation, Methodology, Visualization, Writing – original draft, Writing – review & editing. DD: Data curation, Investigation, Writing – original draft. NO: Data curation, Investigation, Writing – original draft. JZ: Data curation, Investigation, Writing – original draft. RO: Data curation, Formal analysis, Investigation, Writing – original draft. MM: Data curation, Formal analysis, Investigation, Writing – original draft. SPS: Data curation, Formal analysis, Investigation, Writing – original draft. TK: Data curation, Formal analysis, Investigation, Writing – original draft. TL: Data curation, Resources, Writing – original draft. SS: Data curation, Resources, Writing – original draft. MK: Data curation, Resources, Writing – original draft. RA: Conceptualization, Investigation, Methodology, Project administration, Supervision, Visualization, Writing – original draft, Writing – review & editing. HK: Conceptualization, Funding acquisition, Methodology, Project administration, Supervision, Visualization, Writing – original draft, Writing – review & editing. MJ: Conceptualization, Funding acquisition, Methodology, Project administration, Supervision, Visualization, Writing – original draft, Writing – review & editing. NA: Conceptualization, Funding acquisition, Project administration, Supervision, Visualization, Writing – original draft, Writing – review & editing.

## Supplementary material

Supplementary Figure 1 Biochemical characterization of IBT-V02 formulations after storage for 1 and 3 months. **(A, C)** SDS-PAGE analysis of 1µg Lyo IBT-V02 stored at -20°C (Lane 2) and 30°C (Lane 3), 1µg SFD IBT-V02 stored at -20°C (Lane 5) and 30°C (Lane 6), 1µg Liq IBT-V02 stored at 30°C without desorption (Lane 8) and with desorption (Lane 9) and 1µg freshly prepared Liq IBT-V02 without alhydrogel without desorption (Lane 11) and with desorption (Lane 12) after storage for 1 month **(A)** and 3 months **(C)**. (B and D) SEC-HPLC of Lyo (-20°C, 30°C), SFD (-20°C, 30°C), Liq (30°C, ± desorption) and freshly prepared liq (± desorption) after storage for 1 month **(B)** and 3 months **(D)**.

Supplementary Figure 2 Biochemical characterization of the Lyo IBT-V02 formulation after storage for 6, 9, and 12 months. **(A, C, E)** SDS-PAGE analysis of 1µg Lyo IBT-V02 stored at -20°C (Lane 2) and 30°C (Lane 3) and 1µg freshly prepared Liq IBT-V02 without alhydrogel (6mo: Lanes 5, 9; 12 mo: Lane 4) after storage for 6 months **(A)**, 9 months **(C)**, and 12 months **(E)**. **(B, D, F)** SEC-HPLC of Lyo IBT-V02 stored at -20°C and 30°C and freshly prepared Liq without alhydrogel after storage for 6 months **(B)**, 9 months **(D)**, and 12 months **(F)**.

Supplementary Figure 3 Immunogenicity of the Lyo IBT-V02 formulation after long-term storage. Serum toxin neutralizing titers to toxins Hla, PVL, LukAB, SEA, SEB, TSST-1 for individual BALB/c mice (n = 5/group) immunized with 50µg of Lyo IBT-V02 (as described in Methods) on Day 0 of stability study or after 6 months storage of vaccine at either -20°C or 30°C. Bars indicate mean log-transformed ND_50_, the serum dilution that elicits 50% of neutralizing titer. Dotted lines indicate the limit of detection for each toxin (LOD). Statistical analysis performed with the Mann-Whitney test.

Supplementary Figure 4 Toxin neutralization of LMIC supernatants *in vitro*. Toxicity of bacterial supernatants and neutralizing efficacy of IBT-V02 pAbs tested with rabbit red blood cells (RRBCs) **(A)** and induced HL-60 cells **(B)**. Effective dilutions of bacterial supernatants causing 85% toxicity (ED_85_) against RRBCs, and HL-60 cells were calculated for each LMIC isolate. Bacterial SUP dilutions [1:17.3 (KCO075); 1:171 (Pyo603); 1:32.3 (Gaza209)] resulting in ED_85_ were used to determine concentration of IBT-V02 Rb pAb required to neutralize 50% of bacterial SUP toxicity (NC_50_).

Supplementary Table 1 List of PCR targets and primers. List of PCR gene targets and primers for enterotoxins, protein A (Spa), SCC*mec*, and multilocus sequence typing (MLST).

Supplementary Table 2 Characterization of LMIC isolates *in vitro*. Genotyping was conducted on the strains at Marshfield clinic and results reported in the form of a table. (P, Positive; N, Negative; NA, CC not available; RED, New Sequence Type; NT, Non-Typeable).
