# Supplementary figures and images for: Dry and liquid formulations of IBT-V02, a novel multi-component toxoid vaccine, are effective against Staphylococcus aureus isolates from low-to-middle income countries
Source: Front Immunol. 2024 Apr 3;15:1373367. doi: 10.3389/fimmu.2024.1373367 (PMC11022162; doi:10.3389/fimmu.2024.1373367)

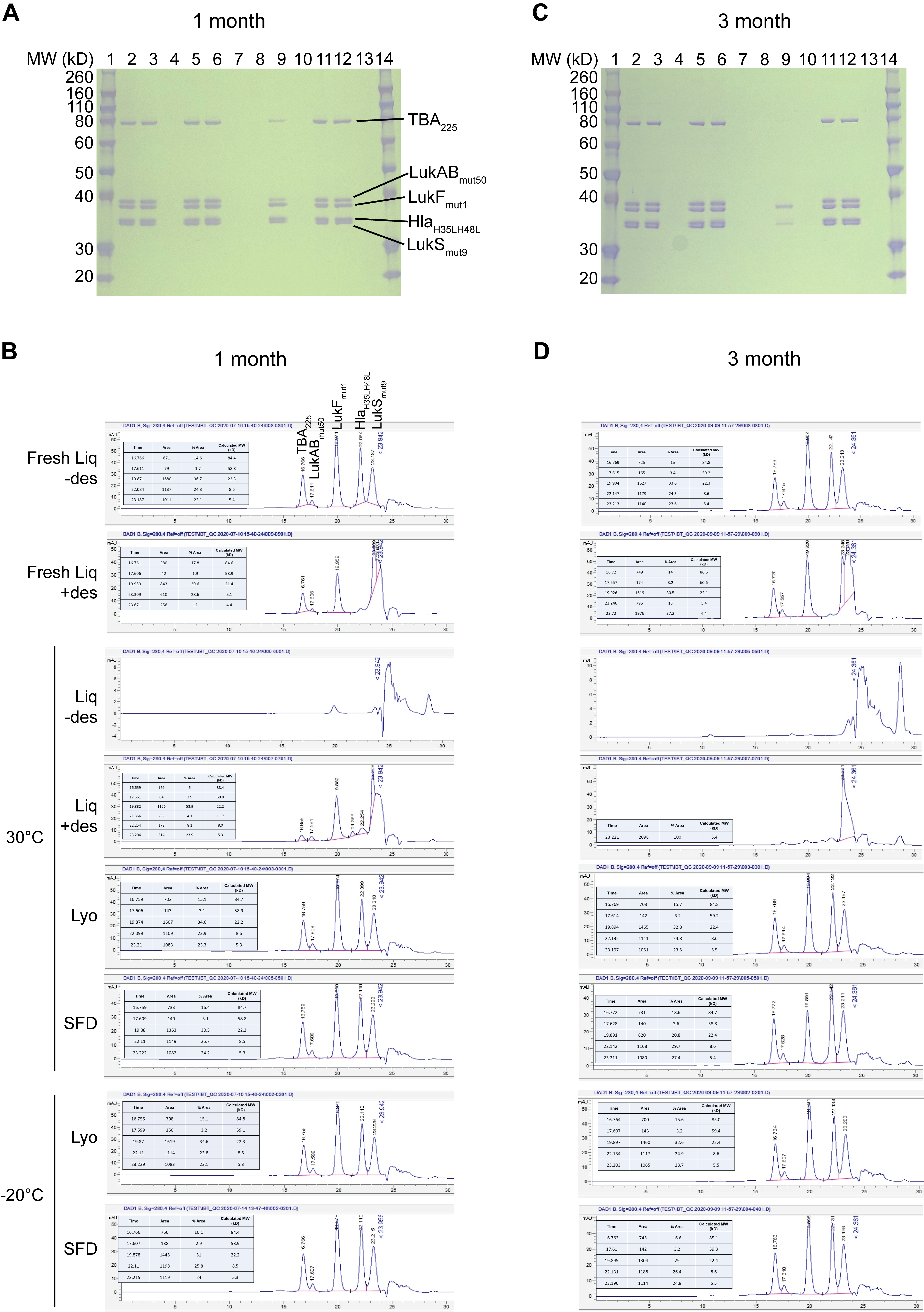

Supplement: Supplementary Figure 1 — Biochemical characterization of IBT-V02 formulations after storage for 1 and 3 months. (A, C) SDS-PAGE analysis of 1µg Lyo IBT-V02 stored at -20°C (Lane 2) and 30°C (Lane 3), 1µg SFD IBT-V02 stored at -20°C (Lane 5) and 30°C (Lane 6), 1µg Liq IBT-V02 stored at 30°C without desorption (Lane 8) and with desorption (Lane 9) and 1µg freshly prepared Liq IBT-V02 without alhydrogel without desorption (Lane 11) and with desorption (Lane 12) after storage for 1 month (A) and 3 months (C). (B and D) SEC-HPLC of Lyo (-20°C, 30°C), SFD (-20°C, 30°C), Liq (30°C, ± desorption) and freshly prepared liq (± desorption) after storage for 1 month (B) and 3 months (D). [file Image_1.tif]

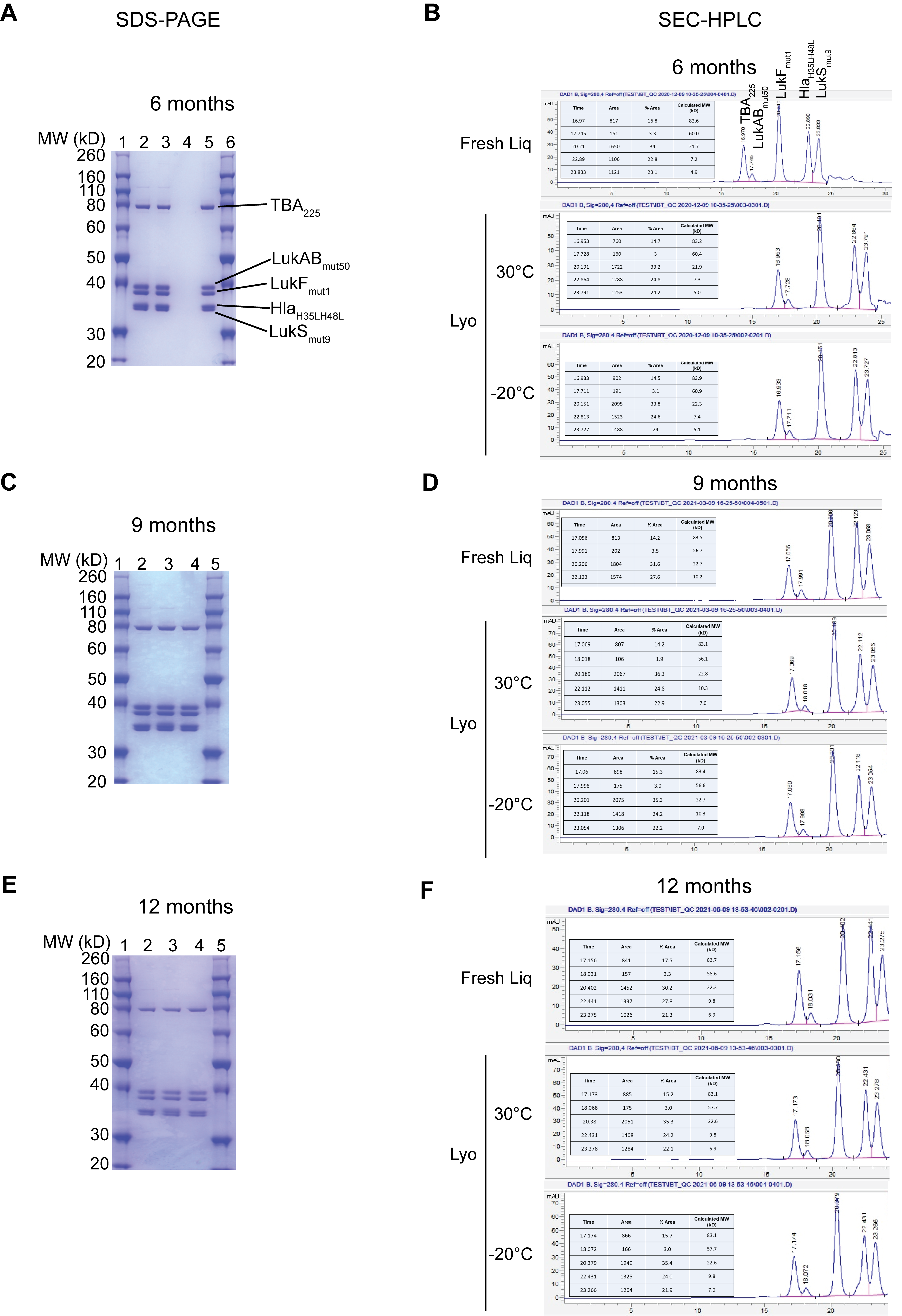

Supplement: Supplementary Figure 2 — Biochemical characterization of the Lyo IBT-V02 formulation after storage for 6, 9, and 12 months. (A, C, E) SDS-PAGE analysis of 1µg Lyo IBT-V02 stored at -20°C (Lane 2) and 30°C (Lane 3) and 1µg freshly prepared Liq IBT-V02 without alhydrogel (6mo: Lanes 5, 9; 12 mo: Lane 4) after storage for 6 months (A), 9 months (C), and 12 months (E). (B, D, F) SEC-HPLC of Lyo IBT-V02 stored at -20°C and 30°C and freshly prepared Liq without alhydrogel after storage for 6 months (B), 9 months (D), and 12 months (F). [file Image_2.tif]

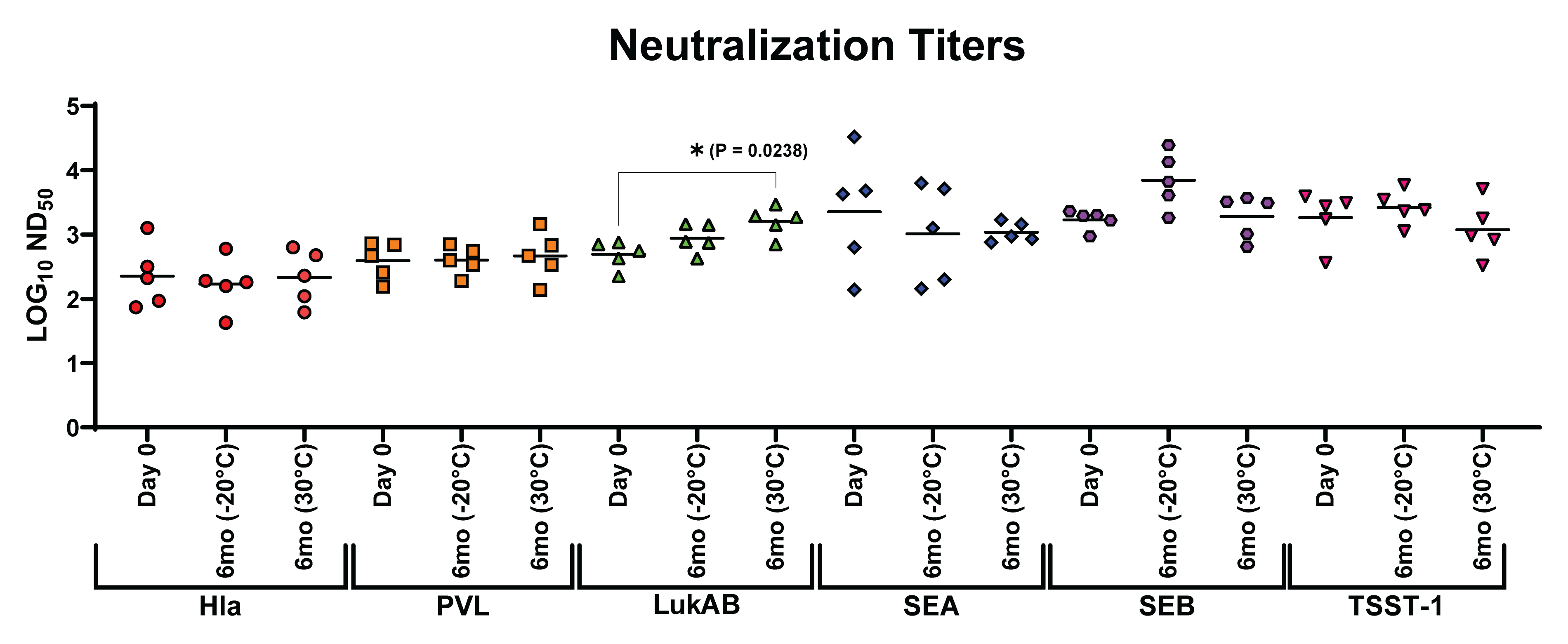

Supplement: Supplementary Figure 3 — Immunogenicity of the Lyo IBT-V02 formulation after long-term storage. Serum toxin neutralizing titers to toxins Hla, PVL, LukAB, SEA, SEB, TSST-1 for individual BALB/c mice (n = 5/group) immunized with 50µg of Lyo IBT-V02 (as described in Methods) on Day 0 of stability study or after 6 months storage of vaccine at either -20°C or 30°C. Bars indicate mean log-transformed ND50, the serum dilution that elicits 50% of neutralizing titer. Dotted lines indicate the limit of detection for each toxin (LOD). Statistical analysis performed with the Mann-Whitney test. [file Image_3.tif]

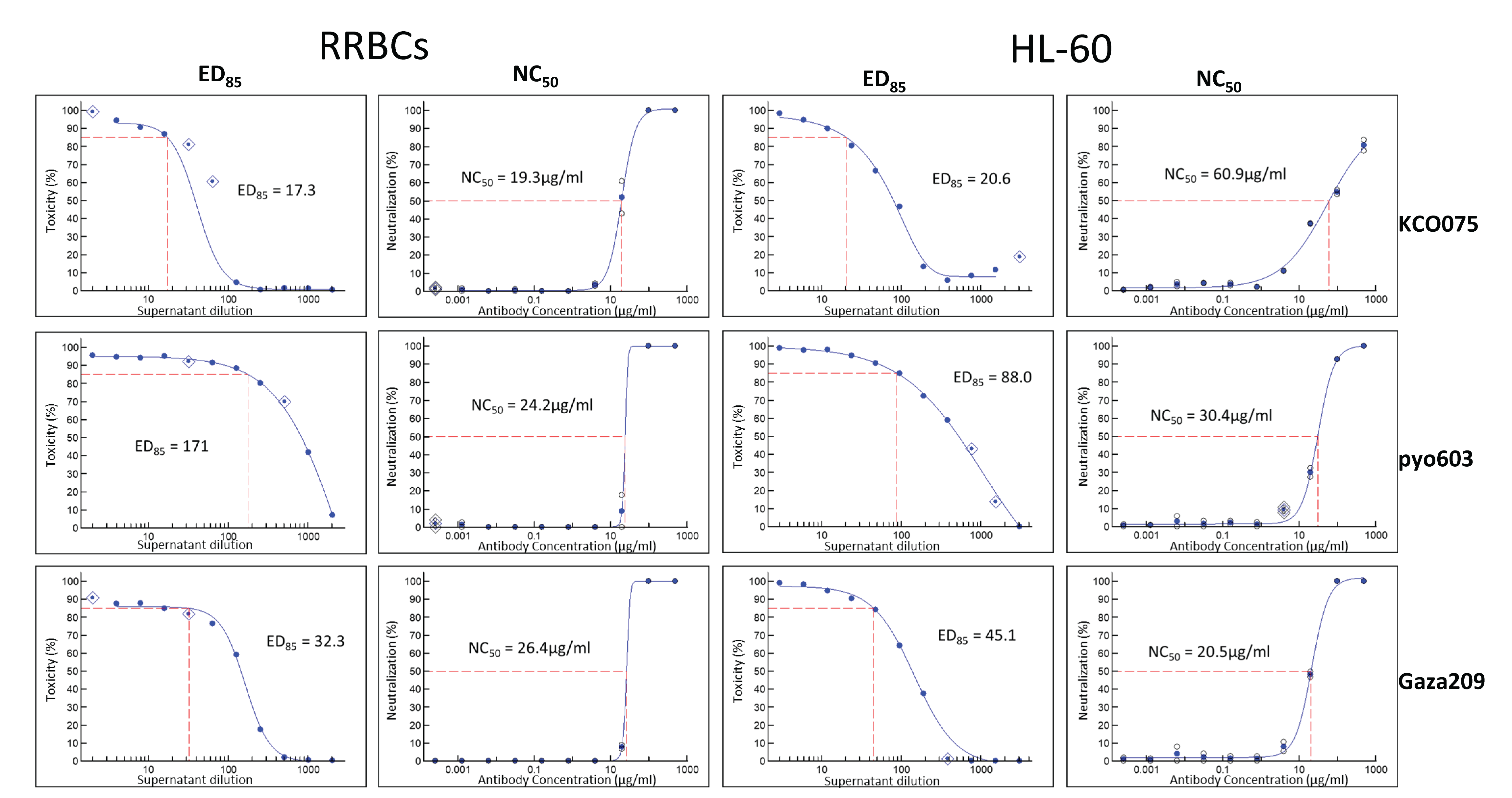

Supplement: Supplementary Figure 4 — Toxin neutralization of LMIC supernatants in vitro. Toxicity of bacterial supernatants and neutralizing efficacy of IBT-V02 pAbs tested with rabbit red blood cells (RRBCs) (A) and induced HL-60 cells (B). Effective dilutions of bacterial supernatants causing 85% toxicity (ED85) against RRBCs, and HL-60 cells were calculated for each LMIC isolate. Bacterial SUP dilutions [1:17.3 (KCO075); 1:171 (Pyo603); 1:32.3 (Gaza209)] resulting in ED85 were used to determine concentration of IBT-V02 Rb pAb required to neutralize 50% of bacterial SUP toxicity (NC50). [file Image_4.tif]
