# Supplementary material for: Dry and liquid formulations of IBT-V02, a novel multi-component toxoid vaccine, are effective against Staphylococcus aureus isolates from low-to-middle income countries
Source: Front Immunol. 2024 Apr 3;15:1373367. doi: 10.3389/fimmu.2024.1373367 (PMC11022162; doi:10.3389/fimmu.2024.1373367)
Supplement: Supplementary Table 1 — List of PCR targets and primers. List of PCR gene targets and primers for enterotoxins, protein A (Spa), SCCmec, and multilocus sequence typing (MLST). [file Table_1.docx]

Supplemental Table 1. List of PCR targets and primers.

| **Target** | **Primer Sequence (5' to 3')** | **Expected PCR Product Size (bp)** |
| --- | --- | --- |
| **Enterotoxin primers** | | |
| SEA-3 | 5' - CCT TTG GAA ACG GTT AAA ACG | 127 |
| SEA-4 | 5' - TCT GAA CCT TCC CAT CAA AAA C |  |
| SEB-1 | 5' - TCG CAT CAA ACT GAC AAA CG | 477 |
| SEB-4 | 5' - GCA GGT ACT CTA TAA GTG CCT GC |  |
| SEC-3 | 5' - CTC AAG AAC TAG ACA TAA AAG CTA GG | 271 |
| SEC-4 | 5' - TCA AAA TCG GAT TAA CAT TAT CC |  |
| SED-3 | 5' - CTA GTT TGG TAA TAT CTC CTT TAA ACG | 319 |
| SED-4 | 5' - TTA ATG CTA TAT CTT ATA GGG TAA ACA TC |  |
| SEE-3 | 5' - CAG TAC CTA TAG ATA AAG TTA AAA CAA GC | 178 |
| SEE-2 | 5' - TAA CTT ACC GTG GAC CCT TC |  |
| TST-3 | 5' - AAG CCC TTT GTT GCT TGC G | 445 |
| TST-6 | 5' - ATC GAA CTT TGG CCC ATA CTT T |  |
| HLA-F | 5' - CAA TCA AAC CGC CAA TTT TT | 174 |
| HLA-R | 5' - CCT GGC CTT CAG CAT TTA AG |  |
| HLB-F | 5' - TAT CCA AAC TGG GGG CAA TA | 292 |
| HLB-R | 5' - AAC CGC TTT TGA AAA CAT GC |  |
| HLD-F | 5' - TAA TTA AGG AAG GAG TGA TTT CAA TG | 100 |
| HLD-R | 5' - TTT TTA GTG AAT TTG TTC ACT GTG TC |  |
| HLGB-F | 5' - GGC AGA CAA AGC AGT GCA TA | 202 |
| HLGB-R | 5' - CTG CCC AGT AGA AGC CAT TC |  |
| HLGA-F | 5’-­CACAAGACCCAACTGGTCCAGCAGC | 202 |
| HLGA-R | 5’-­GCTAAACGATGTCTTGTCACGTAAGC |  |
| HLGC-F | 5’-­GGAAGCGATATAGAAATTATC | 510 |
| HLGC-R | 5’-­TAAATCGCTATCAAAGGCTG |  |
| LUKD-L | 5' - TTG CAC TGC TTT TGC TAT CG | 675 |
| LUKD-R | 5' - GCA TTT GAT GTG TTG GCA AG |  |
| LUKE-L | 5' - GAT TGC GCC TTT AGC ATC TC | 612 |
| LUKE-R | 5' - GCT GAA CCT GTT GGA CCA TT |  |
| LUK-PV-1 | 5' - ATC ATT AGG TAA AAT GTC TGG ACA TGA TCC A | 433 |
| LUK-PV-2 | 5' - GCA TCA AST GTA TTG GAT AGC AAA AGC |  |
| **Spa Primers** |  |  |
| SPAR1 | 5' - CAG CAG TAG TGC CGT TTG C | 200-600 |
| SPAF2 | 5' - GAA CAA CGT AAC GGC TTC ATC C |  |
|  |  |  |
| **SCCmec PCR Primers** | | |
| TYPE I-F | 5' - GCT TTA AAG AGT GTC GTT ACA GG | 613 |
| TYPE I-R | 5' - GTT CTC TCA TAG TAT GAC GTC C |  |
| TYPE II-F | 5' - CGT TGA AGA TGA TGA AGC G | 398 |
| TYPE II-R | 5' - CGA AAT CAA TGG TTA ATG GAC C |  |
| TYPE III-F | 5' - CCA TAT TGT GTA CGA TGC G | 280 |
| TYPE III-R | 5' - CCT TAG TTG TCG TAA CAG ATC G |  |
| TYPE IVA-F | 5' - GCC TTA TTC GAA GAA ACC G | 776 |
| TYPE IVA-R | 5' - CTA CTC TTC TGA AAA GCG TCG |  |
| TYPE IVB-F | 5' - TCT GGA ATT ACT TCA GCT GC | 493 |
| TYPE IVB-R | 5' - AAA CAA TAT TGC TCT CCC TC |  |
| TYPE IVC-F | 5' - ACA ATA TTT GTA TTA TCG GAG AGC | 200 |
| TYPE IVC-R | 5' - TTG GTA TGA GGT ATT GCT GG |  |
| TYPE IVD-F5 | 5' - CTC AAA ATA CGG ACC CCA ATA CA | 881 |
| TYPE IVD-R6 | 5' - TGC TCC AGT AAT TGC TAA AG |  |
| TYPE V-F | 5' - GAA CAT TGT TAC TTA AAT GAG CG | 325 |
| TYPE V-R | 5' - TGA AAG TTG TAC CCT TGA CAC C |  |
| MECA147-F | 5' - GTG AAG ATA TAC CAA GTG ATT | 147 |
| MECA147-R | 5' - ATG CGC TAT AGA TTG AAA GGA T |  |
| **MLST primers** | | |
| ARCC UP | 5' - TTG ATT CAC CAG CGC GTA TTG TC | 569 |
| ARCC DN | 5' - AGG TAT CTG CTT CAA TCA GCG |  |
| AROE UP | 5' - ATC GGA AAT CCT ATT TCA CAT TC | 535 |
| AROE DN | 5' - GGT GTT GTA TTA ATA ACG ATA TC |  |
| GLPF UP | 5' - CTA GGA ACT GCA ATC TTA ATC C | 575 |
| GLPF DN | 5' - TGG TAA AAT CGC ATG TCC AAT TC |  |
| GMK UP | 5' - ATC GTT TTA TCG GGA CCA TC | 487 |
| GMK DN | 5' - CGCGCTCTCTTTTTAAGTGC |  |
| PTAUP | 5' - GTT AAA ATC GTA TTA CCT GAA GG | 574 |
| PTA DN | 5' - GAC CCT TTT GTT GAA AAG CTT AA |  |
| TPI UP | 5' - TCG TTC ATT CTG AAC GTC GTG AA | 470 |
| TPI DN | 5' - TTT GCA CCT TCT AAC AAT TGT AC |  |
| YQIL UP | 5' - CAG CAT ACA GGA CAC CTA TTG GC | 597 |
| YQIL DN | 5' - CGT TGA GGA ATC GAT ACT GGA AC |  |
